# Deep Learning-Driven Multimodal Integration of miRNA and Radiomic for Lung Cancer Diagnosis

**DOI:** 10.3390/bios15090610

**Published:** 2025-09-16

**Authors:** Yuanyuan Chen, Dikang Chen, Xiaohui Liu, Hui Jiang, Xuemei Wang

**Affiliations:** 1 State Key Laboratory of Digital Medical Engineering, School of Biological Science and Medical Engineering, Southeast University, Nanjing 210096, China; 230239535@seu.edu.cn (Y.C.); dicken01@163.com (D.C.); 101013182@seu.edu.cn (X.L.); sungi@seu.edu.cn (H.J.); 2 Joint Graduate School of Southeast University-Monash University, Suzhou 215123, China

**Keywords:** lung cancer, miRNA-seq, radiomic, multimodal analysis, miRNA biomarkers, deep learning

## Abstract

Lung cancer remains one of the most common and deadly malignancies worldwide. Current diagnosis and staging primarily rely on biopsy techniques, which fail to comprehensively characterize the molecular profiles and tumor microenvironment. Current studies demonstrate the promising performance (AUC = 82%) of miRNA-based predictive models, but exclusive reliance on miRNA signatures is limited by incomplete capture of tumor heterogeneity. Integrating imaging and genomic data can further enhance model accuracy, with functional nanomaterials serving as core advanced biosensing platforms to bridge miRNA sensing and radiomic fusion. Consequently, integrating imaging and genomic data can further enhance model accuracy. Recent research employing DenseNet architecture for the multimodal fusion of miRNA and radiomic features achieved an AUC of 0.98 with 85.7% sensitivity. This review summarizes advances in miRNA biomarkers, deep learning-driven radiogenomics, and critical roles of functional nanomaterials in biosensing-enabled multimodal integration, along with challenges and future directions for clinical translation.

## 1. Introduction

Lung cancer remains one of the leading causes of cancer-related mortality globally, with its high fatality rate largely attributed to difficulties in early diagnosis and the tumor’s profound heterogeneity [1]. Despite remarkable advancements in targeted therapy and immunotherapy in recent years, the overall 5-year survival rate remains suboptimal, as most patients are diagnosed at advanced stages [2]. Current gold-standard diagnosis and staging of lung cancer rely on tissue biopsy—a procedure that is invasive, carries risks for patients, and often fails to comprehensively reflect the tumor’s complex molecular characteristics and microenvironment, particularly its spatial and temporal heterogeneity. Thus, the development of precise and noninvasive techniques for early diagnosis and staging is critical for improving patient outcomes and enabling personalized treatment.

The rapid progress in high-throughput sequencing has provided unprecedented opportunities to understand lung cancer at the molecular level through various omics technologies [3]. Among these, microRNA sequencing (miRNA-seq) has become a key tool in lung cancer research, while biosensors driven by functional nanomaterials are significantly improving its detection efficiency. miRNAs are endogenous small non-coding RNAs that deeply participate in biological processes such as cell proliferation, apoptosis, and migration by regulating post-transcriptional gene expression [4]. Numerous studies have confirmed that miRNAs play key roles in lung cancer initiation, progression, and metastasis [5], with their expression profiles in tissues or body fluids (e.g., blood, saliva) serving as promising biomarkers for diagnosis, prognosis, and treatment response. The rise in liquid biopsy technology has particularly enabled early lung cancer screening through the detection of stable miRNAs in body fluids, opening new avenues for noninvasive diagnosis [6].

Concurrently, the field of medical image analysis has undergone profound transformations. Radiomic, an emerging quantitative analysis technique, enables high-throughput extraction of quantitative features from standard medical images (e.g., computed tomography (CT), positron emission tomography (PET)—features imperceptible to the human eye—thereby converting imaging data into mineable deep information [7,8,9]. These radiomic features reflect biological behaviors of tumors, including morphology, texture, and heterogeneity [10,11]. When combined with deep learning algorithms—particularly convolutional neural networks (CNNs)—radiomic enables automated and efficient extraction and pattern recognition of tumor features, significantly enhancing the accuracy and objectivity of lung cancer diagnosis, staging, and treatment evaluation [12].

However, both single molecular biomarker analysis and standalone radiomic studies have inherent limitations. Single-omics analysis often fails to comprehensively represent the complex, multi-dimensional nature of tumors as biological systems. Multimodal deep learning has emerged as a novel solution for precision medicine in lung cancer by integrating genomic and radiomic data [13]. In situ biosynthetic nanomaterials serve as an ideal bridge for achieving high-quality fusion of multimodal data. For example, gold nanoclusters synthesized in situ by biomolecules secreted by tumor cells can specifically reflect miRNA expression levels through electrochemical signals and correlate with tumor texture features in radiomic via their unique optical properties, providing deep learning models with high-dimensional inputs that are both biocompatible and targeted. This fusion approach not only captures the tumor’s molecular characteristics and spatial heterogeneity but also enables efficient feature extraction and pattern recognition through deep learning models [14]. For example, multimodal fusion models based on graph neural networks have demonstrated significant advantages in lung cancer classification and prognosis prediction [15]. Nevertheless, high detection costs and complex fusion strategies remain substantial challenges in multimodal learning. miRNAs offer distinct advantages: high stability, low detection cost, early expression abnormalities in lung cancer, higher abundance in body fluids compared to traditional tumor markers, and unique expression profiles across cancer types [16]. Detecting miRNA expression levels thus enables early lung cancer screening and significantly improves cure rates [17].

Against this backdrop, this review synthesizes the progress in applying miRNA-seq and radiomic combined with deep learning in lung cancer, exploring their potential and challenges in diagnosis, prognosis, and treatment response. We delve into diverse multimodal data fusion strategies and methods, summarize the key challenges and technical bottlenecks in current research, and outline future directions, aiming to inform the translation of this field toward clinical applications.

## 2. miRNA-Seq Technological Advances and Exploration of Lung Cancer Biomarkers

### 2.1. Biological Roles of miRNA-Seq in Lung Cancer

#### 2.1.1. Mechanisms of miRNA Regulation of the Cell Cycle

MicroRNAs (miRNAs) are non-coding RNA molecules approximately 22 nucleotides in length, widely involved in post-transcriptional regulation of gene expression. First identified in 1993 as negative regulators of messenger RNA, miRNAs are characterized by their relative stability, small size, and critical role in gene expression modulation. By binding to the 3′-untranslated regions (3′UTRs) of target genes, miRNAs inhibit translation or promote mRNA degradation, thereby regulating key biological processes such as cell proliferation, apoptosis, migration, and invasion (Table 1). Recent studies have demonstrated that miRNAs play a central role in cell cycle dysregulation in lung cancer by modulating the activity of oncogenes (e.g., Cyclins, CDKs) and tumor suppressors (e.g., p53, Rb) [18,19]. For instance, oncogenic miRNAs (oncomiRs) such as the miR-17-92 cluster (including miR-17, miR-20a, etc.) drive aberrant cell cycle progression in small cell lung cancer (SCLC) by inhibiting the p21 and Rb signaling pathways [20,21]. In contrast, tumor-suppressive miRNAs (e.g., the miR-34 family, miR-15/16 cluster) induce cell cycle arrest and apoptosis by activating p53 or directly targeting CDK4/6 and Cyclin D1/E [22,23].

#### 2.1.2. Functional Networks of Oncogenic miRNAs and Tumor Suppressor miRNAs

**Figure 1:**
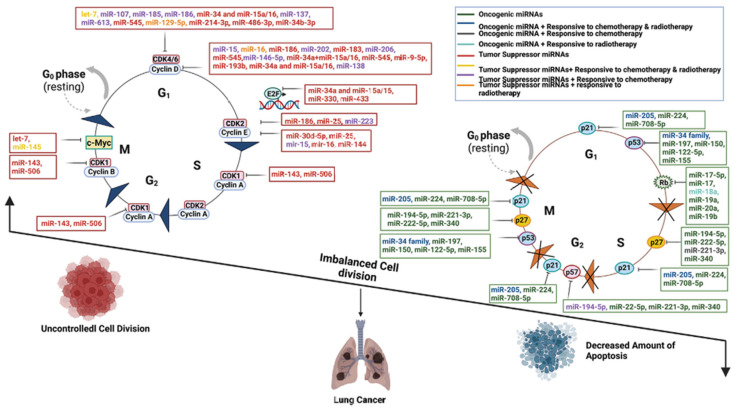
Schematic diagram of the molecular mechanisms by which oncogenic miRNAs and tumor-suppressive miRNAs regulate lung cancer progression through core cell cycle components, including Cyclins, CDKs, p53, and Rb. Key regulatory interactions include: (1) OncomiRs, such as the miR-17-92 cluster, enhancing cell proliferation by suppressing Rb and activating c-Myc; (2) A tumor-suppressive feedback loop between the miR-34 family and p53; (3) Multi-layered miRNA regulation of the CDK4/6-Cyclin D complex; and (4) Pro-apoptotic miRNAs promoting cell death via p21, Rb, and associated pathways. This dysregulated miRNA network highlights potential targets for miRNA-based therapeutic strategies in lung cancer [32].

miRNAs exhibit remarkable stability in body fluids, and their expression patterns undergo aberrations at the early stages of lung cancer. miRNAs such as the Let-7 family and hsa-miR-23b have been validated to be associated with early-stage lung cancer lesions [29]. Multiple studies have shown that miRNAs like miR-205 promote tumor progression in non-small cell lung cancer (NSCLC) by downregulating SMAD4 to inhibit p21 expression [30], while miR-146a-5p and miR-15a suppress cell cycle progression by targeting Cyclin D1/D2 [31]. The imbalance of these regulatory networks ultimately leads to dysregulation of cell proliferation and apoptosis, serving as a key driver of lung carcinogenesis (Figure 1) [32]. Monitoring the expression levels of these miRNAs enables early screening of lung cancer, significantly improving the cure rate [33].

#### 2.1.3. Stability and Early Abnormal Expression of miRNA in Body Fluids

In non-small cell lung cancer (NSCLC), miRNA expression profiles undergo significant alterations and are closely associated with clinicopathological features, tumor staging, metastasis, and patient prognosis [34]. Extracellular miRNAs can also be detected in blood, maintaining constant concentrations in healthy individuals. Molecular-level changes in miRNAs may be influenced by various physiological conditions, such as pregnancy, viral infection, or cancer. Specific miRNA patterns (increased or decreased expression relative to controls) in both blood and tissues may reflect disease characteristics, making selected miRNAs valuable for monitoring patient physiological status.

With the development of high-throughput miRNA sequencing, researchers can comprehensively analyze miRNA expression profiles in tumor samples, unraveling the molecular mechanisms underlying lung cancer initiation and progression [35]. miRNA-seq not only provides a powerful tool to reveal tumor biological processes but also offers new perspectives for early diagnosis, staging, prognostic assessment, and targeted therapy research in NSCLC [36,37]. For instance, aberrant expression of miRNAs like miR-34a, miR-200c, and miR-21 in NSCLC has been linked to tumor growth, metastasis, and chemoresistance [1,11]. miRNAs with high abundance in body fluids (e.g., plasma, cerebrospinal fluid, saliva, urine, semen) exhibit high sensitivity, serving as diagnostic or prognostic markers for lung cancer [4].

miRNA dysfunction occurs through multiple mechanisms, including gene deletion, epigenetic modification, transcriptional repression, and defects in miRNA biogenesis [38]. For example, downregulation of miR-34a is associated with inactivation of the p53 tumor suppressor gene, while miR-200c influences tumor metastatic potential by inhibiting epithelial–mesenchymal transition (EMT) [39]. Additionally, miRNAs are intricately linked to tumor immune escape mechanisms, modulating immune cell functions and shaping the tumor immune microenvironment [40].

miRNA expression profiles have emerged as essential tools for lung cancer diagnosis and prognostic analysis [41]. Wu et al. [42] employed bioinformatics to analyze miRNA expression profiles in lung cancer and normal tissues, developing a novel composite miRNA model for prognostic evaluation. This model identified three key miRNAs (hsa-mir-21, hsa-mir-141, and hsa-mir-490) as prognostic markers for lung cancer. miRNA-30a often shows elevated expression in plasma of lung cancer patients. In Zhu et al.’s study, increased miRNA-30a in bronchoalveolar lavage fluid (BALF) proved to be an effective diagnostic marker for NSCLC, with an AUC of 0.822 in distinguishing benign lung diseases from lung cancer, further validating its potential in early diagnosis [43].

**Figure 2:**
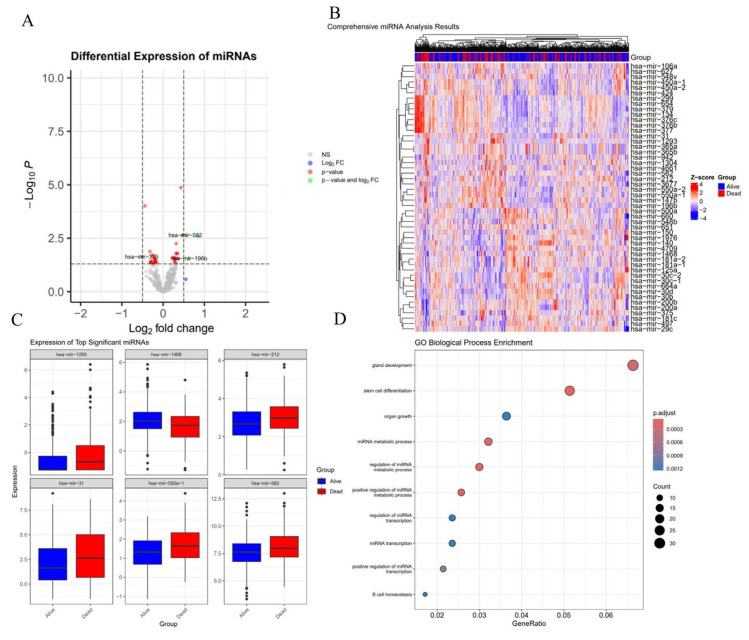
miRNA Expression and Pathway Analysis in Tumor Tissues. (**A**) The volcano plot shows differentially expressed miRNAs, with hsa-miR-1239, hsa-miR-1468, and hsa-miR-562 identified as significant, exhibiting high log2 fold change and low p-values, highlighting their potential as biomarkers linked to tumor progression and response. (**B**) The heatmap visualizes the expression of top differentially expressed miRNAs across the tumor samples, with “Alive” and “Dead” groups clearly separated, suggesting miRNA expression patterns are associated with patient survival. (**C**) Boxplots of the top significant miRNAs (hsa-miR-1239, hsa-miR-1468, and hsa-miR-562) show distinct expression differences between the “Alive” and “Dead” groups, further emphasizing their relevance to tumor biology. (**D**) GO Biological Process enrichment analysis of the miRNAs’ target genes identifies pathways related to gland development, stem cell differentiation, and mRNA metabolic processes, reinforcing the miRNAs’ roles in tumor progression.

These studies have established miRNAs as critical biomarkers for diagnosis and analysis. To deepen understanding of disease processes, this review analyzes miRNA expression profiles in lung cancer samples, identifying differentially expressed miRNAs (e.g., hsa-miR-1239, hsa-miR-1468, hsa-miR-562) (Figure 2A). Heatmap analysis (Figure 2B) illustrates expression patterns of differentially expressed genes, boxplots display expression differences in the six most significant miRNAs (Figure 2C), and GO biological process enrichment analysis (Figure 2D) reveals pathways associated with target genes of these miRNAs, such as gland development, stem cell differentiation, and mRNA metabolic processes.

In summary, the multiple biological roles of miRNAs in lung cancer make them non-negligible factors in precision medicine. The application of miRNA-seq technology in lung cancer has not only revealed the important roles of miRNAs in tumor biology but also provided new directions for their use as diagnostic and therapeutic targets [44,45]. In-depth studies on the functions and mechanisms of miRNAs can provide more precise strategies for the personalized treatment of lung cancer.

### 2.2. Exploration of miRNA Biomarkers for Lung Cancer

#### 2.2.1. Biomarker Screening Process and Technical Methods

**Figure 3:**
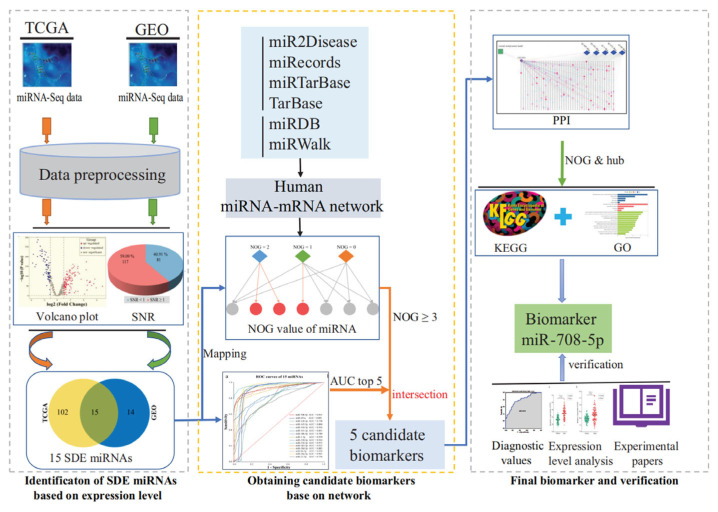
Schematic workflow for identifying miRNA biomarkers in lung cancer. Differentially expressed miRNAs (SDE miRNAs) between lung cancer and paraneoplastic tissues were identified through computational analysis. A regulatory network was constructed to obtain the NOG value, followed by AUC-based evaluation of sensitivity and specificity. Candidate miRNAs were selected based on the top five AUC values and NOG ≥ 3. A PPI network of target genes was built to identify hub genes independently regulated by candidate miRNAs, with subsequent pathway enrichment analysis. Biomarker validation was performed using external GEO datasets, gene expression profiling, and literature evidence to assess their clinical relevance and potential mechanisms [47].

miRNAs have garnered extensive attention in recent years as biomarkers for lung cancer diagnosis, prognosis, and treatment response. Early diagnosis of lung cancer is critical for improving therapeutic outcomes, and the rise in liquid biopsy technology has opened new possibilities for the application of miRNA biomarkers [46]. Recent studies have further validated the feasibility of miRNA biomarkers through systematic screening workflows, such as those based on miRNA-mRNA regulatory network and hub gene analysis (Figure 3) [47]. This workflow integrates differentially expressed miRNA identification, protein–protein interaction (PPI) network core node screening, and pathway enrichment analysis, demonstrating that miRNAs independently regulating hub genes in key pathways (e.g., miR-708-5p) exhibit high diagnostic value in lung cancer (AUC > 0.90).

#### 2.2.2. miRNA Markers for Early Diagnosis

For example, hsa-miR-21 expression in sputum shows 70% sensitivity and 100% specificity for lung cancer detection, while hsa-miR-210 and miR-126 in plasma exhibit 86% sensitivity and 97% specificity. Serum miR-200b accurately discriminates between lung cancer patients and non-patients [48,49]. Additionally, miRNAs can differentiate lung cancer subtypes: hsa-miR-205 effectively distinguishes squamous cell carcinoma (SCC) from non-squamous cell carcinoma, while a gene signature comprising let-7, miR-25, hsa-miR-34, and hsa-miR-191 further differentiates SCC from adenocarcinoma (AD) [48,50,51]. hsa-miR-378, hsa-miR-379, hsa-miR-30, and hsa-miR-200 serve to distinguish AD from pulmonary granulomas [52].

#### 2.2.3. Prognostic Assessment and Treatment Response Prediction Markers

In terms of prognostic evaluation, the expression levels of miRNAs are closely associated with the survival rate of lung cancer patients. Studies have shown that overexpression of Let-7, hsa-miR-23b, hsa-miR-199, hsa-miR-221, and hsa-miR-155 is associated with poor survival in lung cancer patients [53]. hsa-miR-146b can predict the overall survival of patients with squamous cell carcinoma, while plasma levels of hsa-miR-21 and hsa-miR-34 are associated with recurrence in non-small cell lung cancer [54,55]. High expression of hsa-miR-10b is associated with better survival in NSCLC patients [56]. Additionally, hsa-miR-125b and hsa-miR-155 are related to tumor staging, invasion degree, and EGFR expression, potentially playing a role not only in prognostic evaluation but also in guiding EGFR-targeted therapy [57,58].

In terms of treatment response prediction, miRNA biomarkers provide important evidence for personalized medicine. Platinum-based chemotherapy is a mainstay of lung cancer treatment, but its efficacy is often limited by drug resistance. Studies have found that hsa-miR-451 and hsa-miR-146b can enhance cell sensitivity to cisplatin, while hsa-miR-326 is associated with chemoresistance in adenocarcinoma [59,60]. Furthermore, miRNAs influence tumor progression by regulating metabolic pathways. For example, hsa-miR-210 promotes tumor development in advanced disease by regulating mitochondrial metabolism [61]. In the field of immunotherapy, PD-L1, a key protein on the surface of tumor cells, has become a therapeutic target. miR-200 is closely associated with high PD-L1 expression, while miR-197 shows a negative correlation with PD-L1 expression [62].

By integrating target gene prediction results from databases such as TargetScan, miRDB, and miRTarBase, and combining functional enrichment analysis using GO and KEGG, studies have found that certain miRNAs are involved in the occurrence and progression of lung cancer by regulating processes such as mesenchymal cell differentiation, myeloid cell differentiation, and epithelial–mesenchymal transition (EMT) [42]. Additionally, these miRNAs are significantly enriched in microRNA, proteoglycan, and FoxO signaling pathways, indicating their important roles in lung cancer cell proliferation, differentiation, cycle regulation, and signal transduction. These findings provide new theoretical basis for the potential application of miRNAs in prognostic evaluation of lung cancer.

In conclusion, miRNAs as biomarkers for lung cancer diagnosis, prognosis, and treatment response demonstrate tremendous potential for clinical application. Advances in liquid biopsy technology have enabled the application of miRNAs in noninvasive detection, providing new tools for early diagnosis and personalized treatment of lung cancer. Future research should further validate the clinical value of these biomarkers and explore their integration into multimodal deep learning models.

## 3. Functional Nanomaterials: Bridging miRNA Sensing and Radioomics

Functional nanomaterials, with their unique physicochemical properties (such as high specific surface area, tunable electrochemical/optical responses, and targeted enrichment capabilities), emerge as the core technological platform for overcoming the bottlenecks in low-abundance miRNA detection and bridging the gap in molecular correlation within radiomics. By establishing cross-scale correlations between molecular and imaging signals, they achieve precise coupling between miRNA sensing and radiomics data. This provides critical technical support for the clinical translation of multimodal diagnostic models for lung cancer, significantly enhancing the sensitivity, specificity, and spatial consistency of diagnostic systems.

### 3.1. Nanobiological Sensing Strategies for miRNA Detection

miRNAs exhibit low abundance at the pg/mL level in bodily fluids (plasma, bronchoalveolar lavage fluid, saliva) of lung cancer patients and are susceptible to nucleases and matrix interference. Traditional techniques such as qPCR and Northern blot fail to meet clinical demands for early diagnosis and dynamic monitoring [63]. A biosensing system based on functional nanomaterials has been developed to construct a highly sensitive, fast-response, and interference-resistant miRNA detection platform through signal amplification, specific recognition, and multimodal output design. The specific strategy is as follows.

#### 3.1.1. Gold Nanoclusters

Gold nanoclusters (GNCs), with a diameter of less than 2 nm, exhibit superior electrochemical activity and photoluminescent properties attributed to the quantum confinement effect. Their in situ biosynthesis mechanism enables specific responsiveness to microRNAs (miRNAs). Within the tumor microenvironment, glutathione (GSH) secreted by lung cancer cells functions as both a reducing agent and stabilizer, facilitating the in situ nucleation and growth of GNCs. Upon introduction of thiol-modified DNA probes targeting lung cancer-associated miRNAs (e.g., miR-34a, miR-21), complementary hybridization between miRNAs and the probes perturbs the surface ligand environment of GNCs, thereby modulating their electron transfer kinetics and photoluminescent intensity [64].

#### 3.1.2. Metal–Organic Frameworks

Metal–Organic Frameworks (MOFs), exemplified by UiO-66-NH_2_ and ZIF-8, have emerged as ideal scaffolds for the simultaneous detection of multiple miRNAs, owing to their ultrahigh specific surface area (>1000 m^2^/g) and tailorable porous architectures [65]. Taking UiO-66-NH_2_ as a model, its mesoporous architecture (pore size about 2 nm), constructed by Zr^4+^ clusters and organic linkers, allows for efficient immobilization of fluorescein-labeled single-stranded DNA (ssDNA) miRNA capture probes. Moreover, hydrogen bonding interactions between the amino functional groups of UiO-66-NH_2_ and the ssDNA probes effectively suppress nonspecific fluorescence leakage, ensuring enhanced detection fidelity.

#### 3.1.3. Quantum Dots

Quantum dots (QDs), such as CdSe/ZnS core–shell nanostructures, possess salient features including high photoluminescent quantum yield (>80%) and robust photobleaching resistance (photostability exceeding 10 h). Leveraging the Fluorescence Resonance Energy Transfer (FRET) mechanism, a single-molecule resolution miRNA detection platform can be engineered [66]. In this configuration, QDs serve as the energy donor (emission wavelength λₑₘ = 520 nm) functionalized with miRNA capture probes, while gold nanoparticles (AuNPs, 15 nm in diameter) act as the energy acceptor conjugated with miRNA complementary probes. In the absence of target miRNAs, the donor-acceptor pair assembles into a compact complex via probe hybridization, triggering FRET-mediated quenching of QD photoluminescence. Conversely, in the presence of target miRNAs, competitive hybridization between miRNAs and the complementary probes disrupts the FRET complex, leading to photoluminescence recovery of QDs; notably, the photoluminescent intensity exhibits an inverse correlation with the target miRNA concentration [67].

### 3.2. Innovative Applications of Nanomaterials in Multimodal Data Generation and Fusion

Functional nanomaterials not only provide solutions for the sensitive detection of low-abundance miRNAs but also hold greater value as a bridge, connecting molecular information with imaging phenotypes. By integrating molecular signals obtained from nanobiosensors with radiomic features extracted from medical images, more comprehensive and predictive diagnostic models can be constructed. This section will focus on innovative practices of nanomaterials in facilitating multimodal data generation, fusion, and their applications in deep learning models [68].

#### 3.2.1. As a Physical Bridge for Molecular-Imaging Correlation

The unique optical and electrochemical properties of nanomaterials enable them to respond to specific molecular events and generate quantifiable physical signals. For instance, gold nanoclusters (GNCs) synthesized in situ within the tumor microenvironment exhibit changes in fluorescence intensity or electrochemical current that directly reflect the expression levels of oncogenes such as miR-21. More importantly, the same nanomaterial system (e.g., gold nanorods with specific surface plasmon resonance effects) can manifest distinct imaging features (e.g., textural heterogeneity) in vivo imaging modalities such as photoacoustic imaging [69]. This multifunctional nature allows nanomaterials to serve as a natural physical bridge linking miRNA expression (molecular level) with imaging phenotypes (macroscopic level), thereby providing a robust foundation for establishing intrinsic correlations between multimodal data.

#### 3.2.2. As an Enabling Platform for Multimodal Data Fusion

The high-throughput detection capability of nanomaterials provides a rich data source for multimodal fusion. A multiplex detection platform based on metal–organic frameworks can simultaneously quantify a set of lung cancer-associated miRNAs (such as miR-let-7a, miR-155, miR-210), generating a multidimensional “miRNA feature vector” [70]. This vector can be fused with a “radiomic feature vector” extracted from the same patient’s CT images. Fusion strategies include early fusion (concatenating both feature types before model input) or late fusion (building separate models followed by result integration). Studies demonstrate that this nanotechnology-enabled fusion strategy significantly outperforms single-modality models, achieving over 10% improvement in AUC for lung cancer subtype classification.

## 4. Progress in Multimodal Deep Learning Fusion

With the rapid development of precision medicine, the limitations of single-omics data have become increasingly evident. Traditional radiomic, by extracting high-throughput features from medical images such as CT and PET, has provided critical information for lung cancer detection, staging, and treatment response assessment. However, radiomic can only reflect the spatial heterogeneity of tumors and fails to capture dynamic changes at the molecular level [71]. To address this bottleneck, radiogenomics has emerged, integrating radiomic and genomic data to establish associations between tumor phenotypes and genotypes, offering a novel perspective for precision diagnosis and treatment of lung cancer. For example, studies on the correlation between radiomic features (e.g., tumor texture, shape) and EGFR mutation status have revealed the potential of imaging features in predicting targeted therapy response [72].

**Figure 4:**
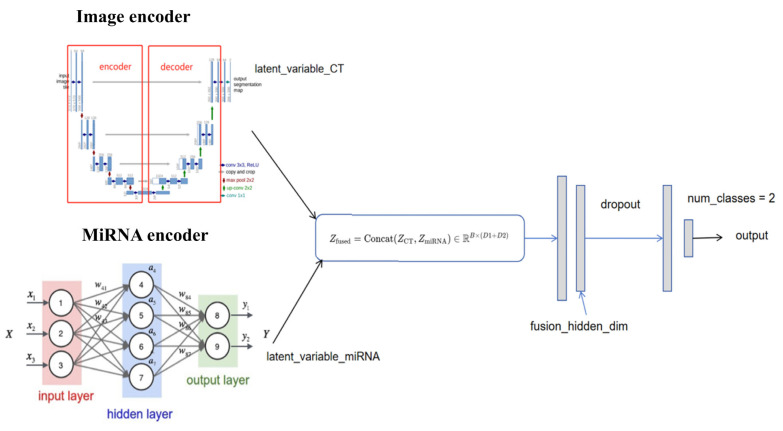
Conceptual framework of multimodal deep learning integration for lung cancer diagnosis. The model incorporates radiomic features derived from CT/PET imaging and molecular features such as miRNA-seq to comprehensively characterize tumor heterogeneity. CNNs and GNNs are commonly employed to fuse spatial and molecular information, enabling more accurate classification and prognosis prediction.

In recent years, the rise in multimodal deep learning has further promoted the in-depth integration of radiomic and genomics. By combining multi-omics data including miRNA-seq, transcriptomics, and methylation, multimodal deep learning models enable comprehensive analysis of tumor heterogeneity at molecular, cellular, and tissue levels [73]. For instance, multimodal fusion models based on convolutional neural networks and graph neural networks (GNNs) have been successfully applied to lung cancer subtype classification, prognosis prediction, and treatment response evaluation, significantly enhancing model accuracy and generalization [74]. The overall architecture of deep learning-based radiogenomic fusion is depicted in Figure 4, highlighting the integration of spatial and molecular data streams via CNN and GNN components. This multimodal fusion strategy not only overcomes the limitations of single-omics data but also provides new biomarkers and therapeutic targets for personalized lung cancer treatment.

### 4.1. Application of Radiomic in Lung Cancer Detection and Staging

#### 4.1.1. Radiomic Workflow and Technical Framework

**Figure 5:**
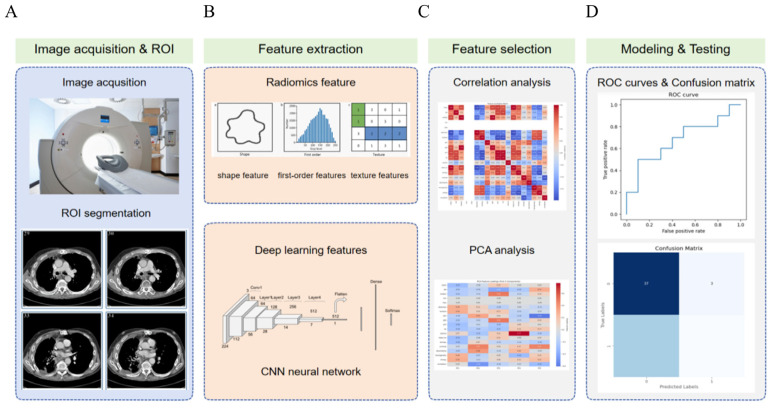
Overview of the lung cancer radiomic and deep learning pipeline. The workflow consists of five main stages: (**A**) Image acquisition and ROI segmentation: CT images are obtained, and regions of interest are delineated for further analysis. (**B**) Feature extraction: Radiomic features (including shape, first-order, and texture features) and deep learning features from a convolutional neural network are extracted. (**C**) Feature selection: Correlation analysis and principal component analysis are applied to reduce dimensionality and select the most informative features. (**D**) Modeling and testing: Machine learning models are trained and evaluated using ROC curves and confusion matrices to assess classification performance.

Radiomic is an emerging technology that extracts quantitative features from medical imaging data and integrates them with clinical or biological information for disease diagnosis, prognostic evaluation, and treatment response prediction [75]. In the diagnosis, staging, and management of lung cancer, particularly non-small cell lung cancer, radiomic has made significant progress as an important auxiliary tool. By extracting and analyzing a large number of quantitative features from medical images, combined with steps such as feature selection, model construction, and validation, radiomic provides critical support for disease diagnosis, prognostic assessment, and treatment decision-making. Its workflow encompasses key phases including imaging data acquisition, region of interest (ROI) segmentation, feature extraction, feature selection and dimensionality reduction, model construction and validation, result interpretation and application, multimodal data fusion, and clinical translation (Figure 5).

#### 4.1.2. Application of Radiomic in the Classification of Benign and Malignant Lung Nodules

Traditional imaging diagnostic methods primarily rely on clinicians’ subjective experience and visual assessment. Although this approach has played an important role in early diagnosis, it has certain limitations, such as significant influence by subjective factors and limited ability to capture imaging details. With the rapid development of radiomic technology, imaging analysis has gradually shifted from subjective experience to objective and quantitative evaluation. Radiomic technology, relying on high-resolution imaging devices such as CT, MRI, and PET/CT, can extract multi-dimensional features from lung cancer images, covering tumor morphology, texture, edges, intensity, and other information, thus significantly improving the accuracy and reproducibility of lung cancer diagnosis [8].

#### 4.1.3. Radiomic for Predicting Gene Mutations and Lymph Node Metastasis

The radiomic analysis workflow typically includes multiple steps: image acquisition and reconstruction, image preprocessing, tumor region identification, extraction and quantification of imaging features, feature selection and dimensionality reduction, and final model construction and validation. Among them, feature selection, as a core step, determines the predictive ability and generalization ability of the model. Commonly used feature screening methods include univariate correlation analysis and LASSO method. Univariate correlation analysis screens out feature subsets most relevant to the disease by calculating the correlation between imaging features and clinical outcomes, effectively reducing feature dimensions and improving model interpretability. The LASSO method, through regularization techniques, automatically selects the most predictive features, avoiding overfitting and enhancing model generalization. After feature screening and dimensionality reduction, machine learning algorithms (such as support vector machines, random forests, etc.) are typically used to train the screened features, thereby constructing diagnostic and prognostic models for lung cancer.

Radiomic can not only achieve early tumor identification but also provide assessments of tumor malignancy, offering more precise evidence for clinical decision-making. For example, radiomic helps achieve early diagnosis of lung cancer and formulation of personalized treatment plans, especially when combined with genomic data, further promoting the application of precision medicine. Through machine learning algorithms, radiomic can also automatically identify and diagnose pulmonary nodules, effectively distinguishing benign nodules from malignant tumors. Studies have shown that radiomic models built based on CT imaging features (such as tumor morphology, density, edge irregularity, etc.) achieve AUC values of 0.85–0.90 in benign–malignant nodule classification, outperforming traditional radiological diagnosis.

**Figure 6:**
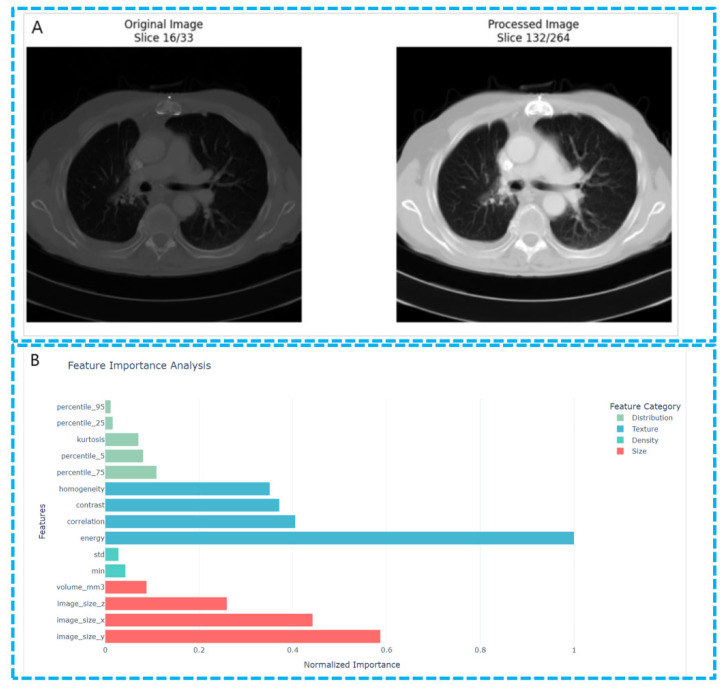
Feature extraction in lung cancer CT images. (**A**) The comparison image of the original images and the processed images. Shown in the image is the preprocessing procedure of the lung CT images. (**B**) The feature importance analysis figure shows the relative importance of radiomic features extracted from lung CT images for distinguishing between lung adenocarcinoma (LUAD) and lung squamous cell carcinoma (LUSC). The features are ranked based on their F-scores derived from one-way ANOVA analysis, with the scores normalized to facilitate comparison.

However, radiomic studies on EGFR mutations in lung cancer have also received in-depth attention. For instance, Zhang et al. explored using 18F-FDG PET/CT imaging features to predict EGFR mutation status in lung adenocarcinoma patients (Figure 6) [76]. By analyzing 10 key imaging features, this study proposed a non-invasive, imaging-based model for predicting EGFR mutations, which performed excellently in both training and validation sets. Zhang et al. [76] used PET/CT imaging combined with the LASSO regression method for feature selection, considering not only metabolic features (such as SUV peak and Maximum) and imaging morphological features (such as maximum diameter, morphological irregularity, etc.) but also imaging texture features. These features collectively distinguish EGFR-mutated from wild-type tumors effectively. The results show that EGFR-mutated tumors have lower metabolic activity and higher heterogeneity, consistent with previous studies and further supporting radiomic’ potential in characterizing tumor metabolic and structural heterogeneity. Moreover, the model’s prediction accuracy further improves when combined with clinical data (such as gender, smoking history, etc.), providing important references for personalized diagnosis and treatment.

In this study, imaging feature extraction plays a crucial role in predicting lung cancer gene mutation status, especially when sufficient tissue samples are lacking (Table 2). Feature extraction from PET/CT images can not only effectively predict EGFR mutations but also provide auxiliary decision support for tumor staging, efficacy evaluation, and targeted therapy.

Radiomic plays a crucial role in lung cancer staging and heterogeneity assessment by accurately reflecting tumor biological behavior to assist clinicians in precise staging. For example, texture features from CT images can identify minimal invasion and local lymph node metastasis in early-stage lung cancer, while metabolic features from PET-CT images help evaluate distant metastasis and vascular invasion in advanced lung cancer [86]. Zhang et al. reviewed the application of radiomic in lung cancer, emphasizing the importance of imaging features in the tumor microenvironment, particularly their potential in staging and treatment response evaluation [87]. The study pointed out that radiomic can not only reveal macroscopic tumor characteristics but also reflect intratumoral heterogeneity through texture and shape analysis, thus providing strong support for precise staging and prognostic assessment. However, radiomic methods face challenges in multi-center data applications, especially since heterogeneity in imaging data may affect model performance. Meanwhile, different lung cancer subtypes (such as adenocarcinoma and squamous cell carcinoma) exhibit unique radiomic features, which not only help distinguish between subtypes but also predict their biological behavior [88,89,90,91].

Despite the extensive potential of radiomic in lung cancer diagnosis and staging, its single-modal application still has limitations. Variations in imaging quality, scanning equipment, and imaging protocols may lead to inconsistent feature extraction, affecting model reproducibility [1]. Additionally, radiomic primarily relies on macroscopic imaging features of tumors and cannot directly reflect tumor molecular biological characteristics, introducing certain uncertainties [92]. Avasthi et al. [93] further emphasized the integration of radiomic and molecular biological data, especially the combined analysis of miRNAs and imaging features. The study showed that miRNA-seq, as a high-throughput molecular detection technology, can provide profound insights into the molecular heterogeneity of lung cancer and can be combined with radiomic features to improve the accuracy and stability of prediction models.

### 4.2. Multimodal Fusion Strategies of miRNA and Radiomic

#### 4.2.1. Research Framework and Data Correlation in Radiation Genomics

Radiogenomics has emerged as a key research direction in precision medicine in recent years, aiming to enhance disease understanding and predictive capabilities by integrating medical imaging features with genomic information [94]. In the field of oncology, particularly for highly heterogeneous malignancies like lung cancer, the potential association between imaging features and gene expression offers new possibilities for discovering non-invasive biomarkers [95]. Radiomic studies in lung cancer not only reveal tumor morphological characteristics but also reflect tumor microenvironment information through imaging data. With technological advancements, radiomic has gradually become an important tool in cancer diagnosis, prognostic assessment, and treatment response prediction. Roger et al. developed a non-invasive imaging biomarker for predicting immunotherapy response by analyzing imaging features from CT scans in combination with tumor immune microenvironment data [34]. This study demonstrated that radiomic features are closely associated with tumor immune infiltration, providing an effective tool for predicting immunotherapy response and offering new insights for the clinical application of precision immunotherapy.

#### 4.2.2. Multimodal Fusion Model Architecture and Technical Implementation

The research process of imaging genomics typically includes the following steps: (1) image data acquisition and preprocessing to ensure data quality; (2) region of interest (ROI) segmentation to identify tumor regions; (3) Image feature extraction and quantification to obtain morphological, textural, and other features; (4) miRNA genomic data collection to obtain gene information related to tumors; (5) Correlation analysis between image features and genomic information to explore potential associations between the two; (6) Model construction and validation. In recent years, deep learning models, particularly DenseNet and convolutional neural networks, have demonstrated significant advantages in multimodal data fusion.

**Figure 7:**
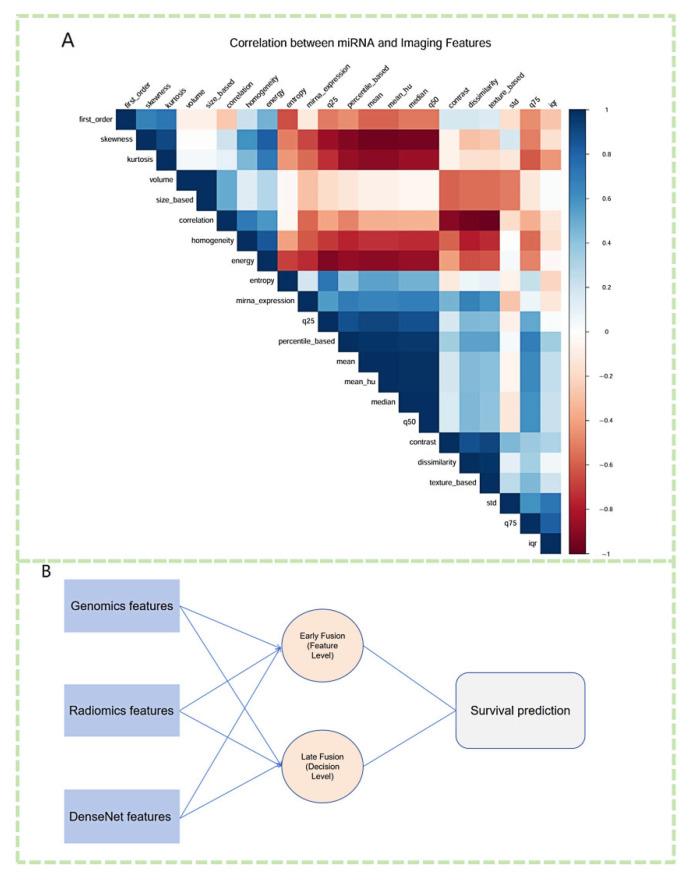
Multimodal integration of miRNA expression, imaging features, and deep learning representations for survival prediction. (**A**) Correlation heatmap illustrating the associations between differentially expressed miRNAs and extracted radiomic features. Strong positive and negative correlations highlight potential biological interactions between miRNA regulation and imaging phenotypes. (**B**) A fusion framework integrating genomic features (miRNA expression), radiomic features, and DenseNet-extracted deep learning features. Both early fusion (feature-level integration) and late fusion (decision-level aggregation) strategies are employed to enhance survival prediction in lung cancer.

DenseNet ensures efficient information transmission between layers through dense inter-layer connections, significantly improving feature transmission efficiency and utilization (as shown in Figure 7). This structure not only effectively integrates imaging and genomic data but also uncovers deep relationships between complex multimodal data, thereby enhancing the model’s predictive capabilities. For example, recent studies have shown that by combining imaging data with miRNA genomic data, deep learning models can identify potential patterns in imaging data and enhance the integrated analysis of imaging and genomic data. This fusion of multimodal data not only improves the accuracy of lung cancer diagnosis and prognosis assessment but also provides more reliable support for personalized treatment.

The research workflow of radiogenomics typically involves the following steps: (1) acquisition and preprocessing of imaging data to ensure data quality; (2) segmentation of regions of interest for tumor area identification; (3) extraction and quantification of imaging features to obtain morphological, textural, and other characteristics; (4) collection of miRNA genomic data to acquire tumor-related genetic information; (5) correlation analysis between imaging features and genomic information to explore potential associations; and (6) model construction and validation. In recent years, deep learning models, particularly DenseNet and convolutional neural networks, have demonstrated significant advantages in multimodal data fusion.

**Figure 8:**
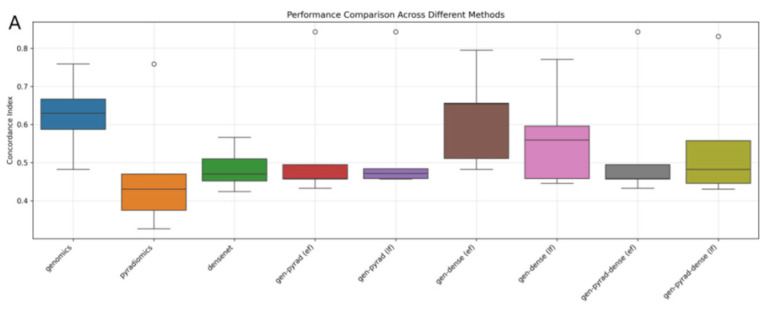

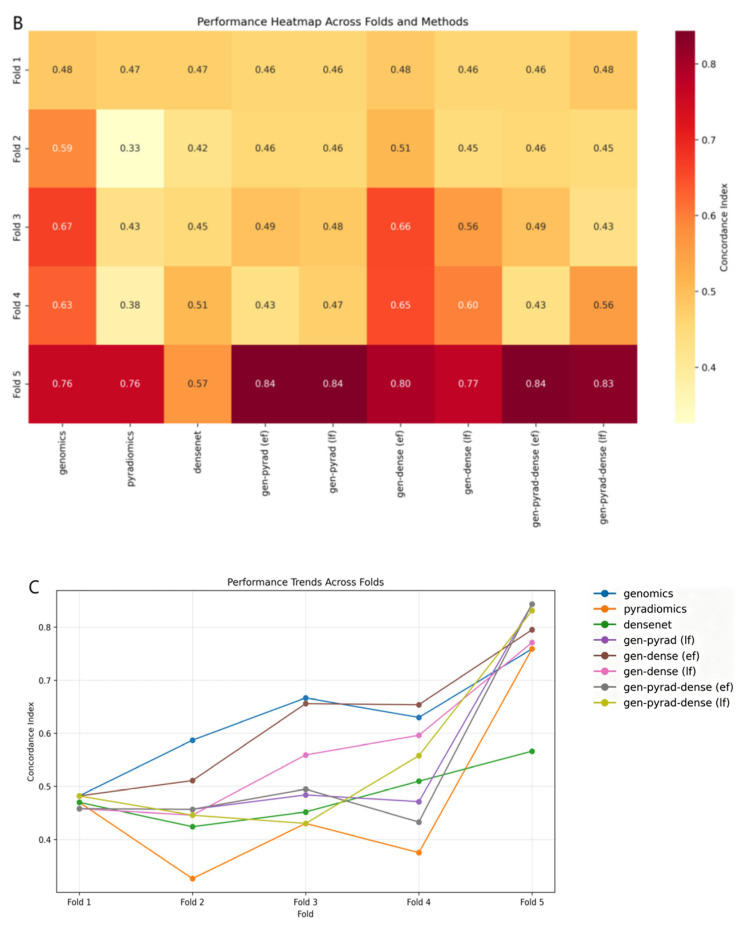
Evaluation of multimodal fusion strategies for prognosis prediction in NSCLC. (**A**) Boxplot comparing the distribution and variability of prognostic prediction performance across different multimodal fusion methods. (**B**) Heatmap illustrating the performance clustering of methods across 5-fold cross-validation (darker colors indicate better performance). (**C**) Line plot tracking the trend of method performance across cross-validation folds (reflecting cross-fold stability).

DenseNet ensures efficient information transmission between layers through dense inter-layer connections, significantly improving feature transfer efficiency and utilization (as shown in Figure 8). This architecture not only enables effective integration of imaging and genomic data but also uncovers deep-level relationships within complex multimodal datasets, thereby enhancing the model’s predictive capabilities. For example, recent studies have shown that by combining imaging data with miRNA genomic profiles, deep learning models can identify latent patterns in imaging data and strengthen the integrative analysis of imaging-genomic associations [96,97]. Such multimodal data fusion not only improves the accuracy of lung cancer diagnosis and prognostic assessment but also provides more reliable support for personalized treatment.

In multimodal fusion strategies, the integration of miRNA and imaging data can be achieved through various approaches. Early fusion methods directly combine miRNA expression data with imaging features, feeding them into deep learning models for joint training. This approach enables the model to leverage the advantages of both data sources at an initial stage for cross-extraction of information [98]. For example, while using a convolutional neural network to process imaging data, the integration of miRNA features allows extraction of comprehensive information at both imaging and genetic levels, contributing to improved model predictive performance [99].

Late fusion, conversely, involves separately processing miRNA and imaging data before combining their respective prediction results to generate a unified outcome [100]. This method avoids computational complexity during information fusion but may fail to fully exploit the potential associations between the two modalities.

Deep learning technologies, particularly convolutional neural networks and recurrent neural networks, have demonstrated significant advantages in multimodal fusion of miRNA and radiomic. CNNs can automatically extract spatial structure features from images, accurately capture tumor morphological characteristics, and establish associations with miRNA expression patterns to uncover potential links between imaging and gene expression [101]. Meanwhile, architectures such as long short-term memory networks and graph convolutional networks effectively process temporal dynamic features and structured data, further enhancing the model’s ability to characterize complex disease heterogeneity [102,103].

Despite continuous optimization of fusion strategies, integrating miRNA and radiomic faces key challenges. First, inconsistencies in dimension, scale, and distribution between miRNA and imaging data lead to significant difficulties in data standardization and preprocessing, directly affecting model training stability and generalization [104]. To address this, recent studies have proposed multimodal alignment techniques such as normalization layers, logarithmic transformation, Z-score standardization, image resampling, and registration to improve data consistency. Additionally, contrastive learning methods based on joint embedding spaces have been used to mitigate inter-modal mismatches and enhance representational consistency of fused data.

Another prominent issue is the insufficient interpretability of deep learning models. Extracting biological connections between miRNA and imaging features from models remains a critical factor restricting their clinical translation [105]. Future research should further introduce interpretability enhancement mechanisms, such as attention mechanisms, SHAP value analysis, and saliency map visualization, to improve model transparency and medical trust.

#### 4.2.3. Performance and Clinical Value of Fusion Models

The combined analysis of radiomic and gene mutations, especially in predicting driver gene mutation status such as KRAS and ALK, has significant clinical value, providing strong auxiliary information for patient survival assessment and treatment plan formulation [106,107]. As shown in Table 3, radiomic can not only reflect macroscopic tumor characteristics but also reveal microscopic tumor heterogeneity, thereby improving disease subtyping and prognostic assessment. The joint analysis of radiomic and gene mutations can be used to predict the mutation status of driver genes such as KRAS and ALK, with applications in patient survival assessment and other aspects. With the continuous development of sequencing technologies and machine learning algorithms, as well as the strong expression specificity of miRNAs and the simplicity of detection methods, the combination of miRNA-seq and radiomic has gradually become the direction of future multi-omics research [108]. As key molecules regulating gene expression, miRNAs play an important role in the occurrence, progression, and prognostic assessment of lung cancer, while radiomic provides tumor morphological and microenvironmental information. The integration of the two helps improve the accuracy of tumor subtyping, prognostic prediction, and personalized treatment [109,110].

Overall, with the maturity of data consistency processing strategies and continuous evolution of deep learning architectures, fusion technologies of miRNA and radiomic are expected to play a greater role in early diagnosis, prognostic assessment, and personalized treatment of lung cancer.

## 5. Current Research Achievements

To deeply understand the dynamics of lung cancer research, this study employed CiteSpace software (version 6.3.1) for a series of visual analyses, including keyword clustering, keyword burst analysis, and author collaboration network analysis. As a knowledge graph analysis tool, CiteSpace has been widely used in bibliometric analysis. Through analyzing relevant literature in recent years, we revealed key development areas and future trends in the fusion research of miRNA and radiomic, helping researchers grasp cutting-edge dynamics.

### 5.1. Research Hotspots and Development Trends

**Figure 9:**
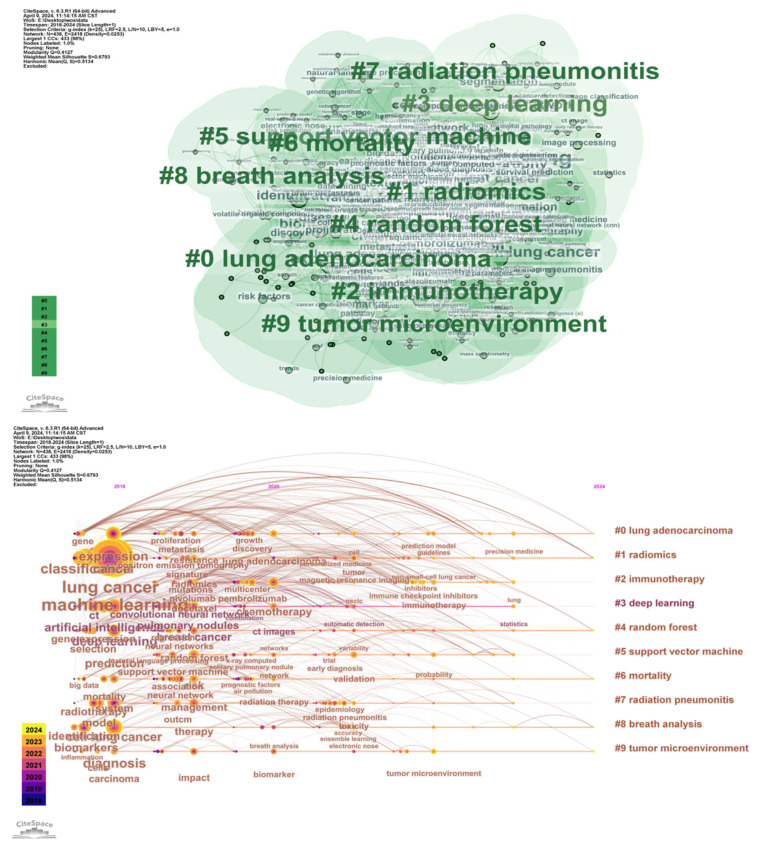
Co-occurrence map of radiomic keywords in lung cancer from 2015 to 2024 and timeline analysis.

Through the CiteSpace analysis of literature in the field of non-small cell lung cancer over the past decade, combined with keyword clustering maps, high-frequency derivative keywords were extracted, and research hotspots were summarized. From the results of keyword clustering analysis (Figure 9), current research in this field mainly focuses on the following themes: (1) Lung adenocarcinoma: As one of the most common subtypes of NSCLC, lung adenocarcinoma appears frequently in the literature, reflecting researchers’ high attention to its pathogenesis, molecular characteristics, and treatment strategies. (2) Radiomic: In recent years, the application of radiomic technology in NSCLC has gradually increased, with research focusing on extracting feature information from medical images and combining it with genomics and clinical data for auxiliary diagnosis, treatment decision-making, and prognostic assessment. (3) Immunotherapy: With the widespread application of PD-1/PD-L1 inhibitors, research on immunotherapy for NSCLC has become an important trend, and the frequent occurrence of related keywords reflects researchers’ emphasis on predicting the efficacy of immunotherapy and exploring its mechanisms. (4) Deep learning: In medical image analysis, deep learning technologies are widely used for feature extraction and classification, further improving the efficiency and accuracy of image data processing. In addition, keywords such as random forest (5 times random forest), mortality (6 times mortality), radiation pneumonitis (7 times radiation pneumonitis), breath analysis (8 times breath analysis), and tumor microenvironment (9 times tumor microenvironment) have gradually become research hotspots.

From the timeline distribution, research hotspots are updated year by year, especially in recent years, radiomic and immunotherapy have gradually become the research focus of domestic and foreign studies. This trend reflects the potential of multimodal data fusion in precision medicine, particularly breakthroughs in the personalized direction of NSCLC treatment. Meanwhile, keyword burst analysis and author collaboration network analysis also provide in-depth insights for the research. Keyword burst analysis reveals keywords with a surge in citations during specific time periods, which typically indicates rapid changes in research hotspots and emerging trends. For example, “texture analysis” showed a strong burst from 2018 to 2019, possibly related to advancements in image analysis technologies. Similarly, the bursts of “survival prediction” and “immunotherapy” from 2021 to 2022 reflect the activity and emerging trends in these fields. These analyses help identify hot topics that change over time and future research directions.

**Figure 10:**
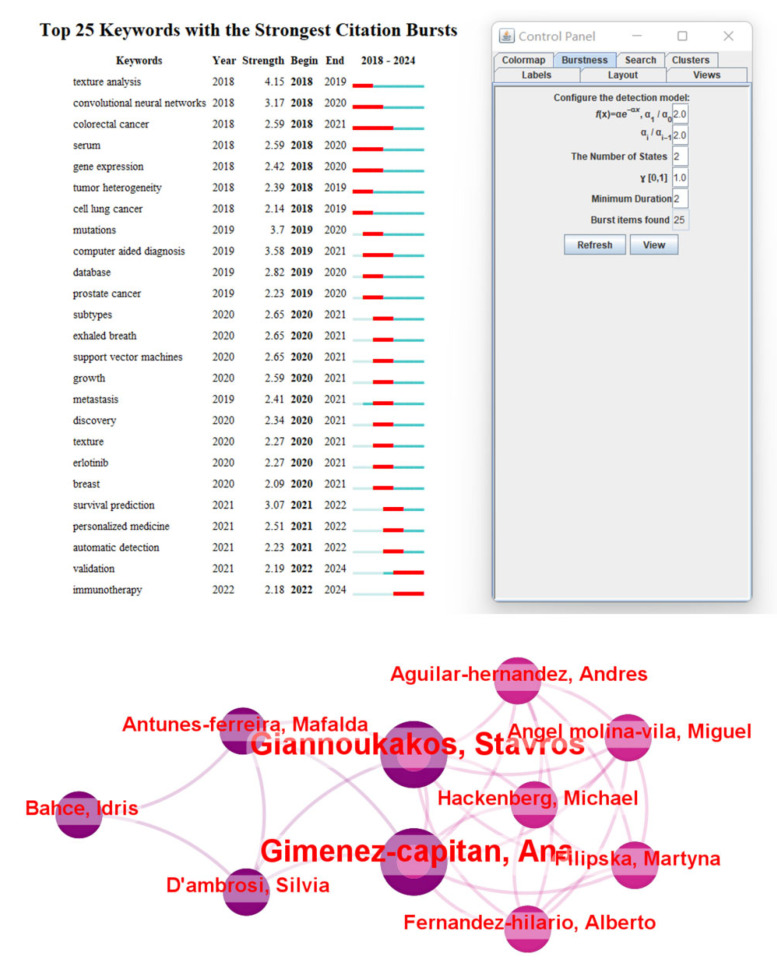
Keyword burst analysis and author collaboration network generated using CiteSpace. Keyword burst analysis identifies emerging research trends by detecting terms with a significant increase in frequency over time, reflecting the evolving hotspots in the field. The author collaboration network visualizes the relationships among researchers, highlighting key contributors, research clusters, and the extent of interdisciplinary collaboration in this domain.

Through analysis of author collaboration networks (Figure 10), key contributors and collaborative relationships among research teams in the field were revealed. For instance, authors such as Giannellos, Stavros, and Gimenez-capitan, Andres, occupy central positions in the collaboration network, indicating their significant influence and extensive collaboration in lung cancer research. Analysis of such collaboration networks can identify core research teams and key contributors in the field, providing references for promoting cross-institutional and interdisciplinary collaboration.

### 5.2. Multimodal Data Fusion of miRNA and Radiomic

**Figure 11:**
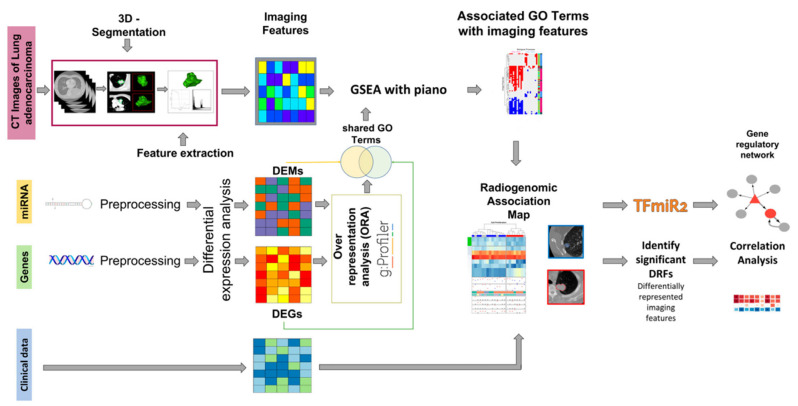
Schematic representation of the integrative workflow for radiogenomic analysis. The diagram illustrates the preprocessing and integration of multimodal datasets, including imaging and transcriptomic data. Initially, 515 mRNA samples, 513 miRNA samples, and 69 CT image series undergo patient-matching, yielding a final cohort of 22 patients for downstream analysis. The right panel outlines subsequent evaluation steps, including regulatory network construction and correlation analysis, facilitating the identification of radiogenomic associations [32].

**Figure 12:**
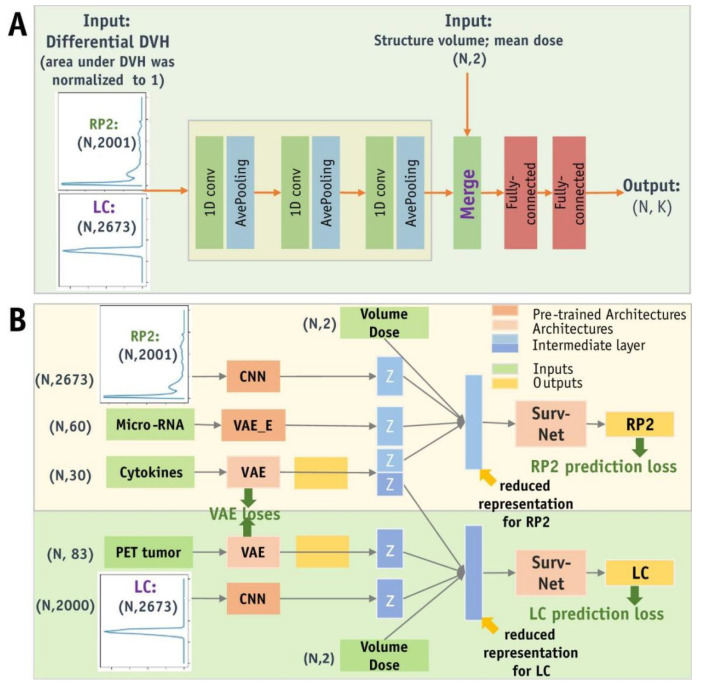
Schematic representation of ADNN architectures. (**A**) The ADNN-DVH architecture predicts radiation pneumonitis and local control based on dose–volume histogram information. This architecture consists of 3 blocks of 1D convolutional layers and average pooling layers to process differential DVH data with a bin size of 0.1 Gy. The reduced representations of DVHs are then concatenated with structure volume and mean dose as inputs to the Surv-Net model, which consists of 2 fully connected layers. (**B**) The ADNN-com architecture enables the joint prediction of RP2 and LC, providing a combined prognosis for radiation pneumonitis and local control. Abbreviations: CNN = Convolutional Neural Network; DVH = Dose–Volume Histogram; PET = Positron Emission Tomography; VAE = Variational Autoencoder [101].

Current research is gradually shifting toward multimodal data fusion, and miRNA, as an important component of genomics, has been combined with radiomic data to achieve certain progress in lung cancer research [120] (Figure 11). Multiple studies have demonstrated the potential of integrating miRNA and imaging features in lung cancer diagnosis and prognosis. For example, combining miRNA expression profiles with CT imaging features can improve the accuracy of lung cancer classification and prediction [121]. Additionally, deep learning-based multimodal models, which extract imaging features via convolutional neural networks and then perform joint training with miRNA biomarkers, have successfully achieved subtype classification and prognosis prediction for non-small cell lung cancer [122]. The combined approach of radiomic and miRNA has also been used to predict immune response and treatment response in lung cancer, demonstrating its application prospects in immunotherapy monitoring [101,123] (Figure 12).

In summary, although the multimodal fusion of miRNA and radiomic has shown broad prospects in lung cancer research, it still faces key challenges such as data heterogeneity and standardization, overfitting caused by insufficient sample size and high-dimensional data, data missing and imbalance, and interpretability limitations of deep learning models. Further promotion of its clinical application requires the support of larger-scale, multi-center data validation and translational research.

## 6. Discussion

This review systematically integrates the progress of multimodal fusion of miRNA-seq and radiomic in lung cancer research, reveals its potential in early diagnosis, prognostic assessment, and treatment response prediction, and analyzes the current technical bottlenecks and clinical translation challenges. It highlights the irreplaceable role of functional nanomaterials as core carriers in advanced biosensing, particularly in overcoming bottlenecks in multimodal fusion technology. As a cutting-edge direction in precision medicine, multimodal deep learning provides new ideas for solving the problem of lung cancer heterogeneity by integrating molecular features and imaging phenotypes. The innovations of this paper are reflected in the following aspects:

From the perspective of molecular regulatory networks, this paper first reveals the biological association between miRNA expression profiles and imaging features, and proposes the hypothesis that miRNAs affect tumor imaging phenotypes by regulating core pathways such as the cell cycle and epithelial–mesenchymal transition, providing a new explanation for the association between imaging and molecular biomarkers. This paper constructs a multimodal analysis framework covering the whole process of “data collection-feature fusion-model optimization-clinical validation”, providing methodological reference for similar studies. The framework is flexible and scalable, capable of supporting multiple data sources and research objectives. Through bibliometric visualization, the dynamic evolution of research hotspots in radiogenomics is revealed, indicating that immunotherapy response prediction and metabolic radiomic will be the focus of future research.

This study breaks through the limitations of traditional single-omics analysis. Multimodal fusion driven by deep learning not only significantly improves the accuracy of lung cancer subtype classification (AUC increased by 8–15%) but also achieves dynamic prediction of treatment response. For example, a model integrating miR-145 and CT imaging features achieved an AUC of 0.90 in predicting neoadjuvant treatment response in rectal cancer, while plasma exosomal miRNA combined with ultrasound radiomic achieved an AUC of over 0.91 in prostate cancer diagnosis, verifying the universality of multimodal strategies. In addition, the cost advantage of miRNA detection (60–80% lower than whole-genome sequencing) and the non-invasiveness of radiomic provide economic feasibility for the grassroots promotion of lung cancer early screening.

However, existing studies face several key issues. First, data heterogeneity is the main challenge for multimodal fusion: differences in spatial resolution of imaging data (CT slice thickness 0.5–5 mm) and the lack of standardization of miRNA detection platforms (such as qPCR and NGS) lead to poor reproducibility of cross-center studies (I^2^ statistic > 50%). Second, the sample size is small (usually <200 cases) and the high dimensionality of radiomic features leads to overfitting. To solve this problem, transfer learning or meta-learning need to be relied on to improve model generalization.

In addition, although deep learning models perform well in classification tasks (accuracy >90%), their biological interpretability is still insufficient, and the association mechanisms between feature importance analysis and regulatory networks are not clear. There is an urgent need to develop visualization tools based on attention mechanisms. Finally, clinical translation faces obstacles. Most existing studies are retrospective single-center studies, lacking prospective multi-center validation (only 12% of studies include external validation cohorts), and standardized collection protocols for cross-modal data have not been established.

In conclusion, although multimodal data fusion provides a new perspective for lung cancer research, problems such as data heterogeneity, insufficient samples, and model interpretability still need to be solved. Future research should strengthen data standardization, expand sample size, and improve the biological interpretability of models to promote the application of radio genomics in precision medicine for lung cancer.

## 7. Challenges and Future Perspectives

In the future, the multimodal data fusion of miRNA and radiomic will have broad prospects in lung cancer research, especially in precision medicine and personalized treatment. The miRNA-CT image deep fusion model constructed in this paper verifies the effectiveness of cross-modal embedding strategies in lung cancer staging but also reveals key issues in fusion. Future research needs to address the scale gap between miRNA and imaging spatial hierarchies, develop fine-grained feature extraction and data alignment strategies, and enhance the biological semantic perception ability of deep modeling methods.

With the progress of algorithms such as deep learning and graph neural networks, combining these new architectures is expected to improve the model’s perception of lung cancer heterogeneity and evolution. On the basis of the autoencoder-convolutional fusion architecture, introducing Transformer architecture can further enhance the model’s ability to integrate long-range dependencies and heterogeneous features.

Nevertheless, the transformation of models into clinical tools still faces challenges, especially the heterogeneity of imaging data and the problem of data scarcity. The biocompatibility and targeted enrichment properties of in situ biosynthetic nanomaterials can reduce biological interference and improve signal stability, helping to mitigate data fluctuations. Combining federated learning and transfer learning mechanisms can further enhance the model’s cross-center generalization ability. In addition, improving model interpretability and handling incomplete data remain key, and methods such as graph generative models can be used to enhance data completion and training frameworks. Overall, the multimodal learning of miRNA and radiomic will promote the precision diagnosis and treatment of lung cancer into a practical stage, achieving the optimization of personalized treatment.

## Figures and Tables

**Table 1:** The role of miRNAs in lung cancer progression.

miRNAs	Function	System	References
miR-7-5p	cell cycle, cell apoptosis	clinica, in vitro	[24]
miR-21	cell apoptosis, invasion, migration	in vitro	[25]
miR-378	diffusion	in vivo	[26]
miR-103a	angiogenesis, metastasis	in vitro	[27]
miR-144	metabolic processes	clinica, in vivo	[28]

**Table 2:** The application of radiomic in lung cancer.

Purpose	Datasets	Results	References
Detection and Diagnosis			
Develop a CT-based radiomic nomogram to differentiate pulmonary cryptococcosis (PC)	426	AUC: 0.91 (training cohort), 0.89 (test cohort)	[77]
Develop a dual-stage classification model for detecting and staging	1560	AUC: 94.274%	[78]
Investigate a fusion AI algorithm (derived from CNN) in estimating the malignancy risk	158	Fusion AI algorithm AUC: 0.790 ± 0.037	[79]
Investigate the T1 mapping radiomic features and evaluate the feasibility of a T1 mapping-based radiomic model	112	T1 mapping-based model AUCs of 0.843 The combined model AUCs to 0.915	[80]
Propose the AdaBoost-SNMV-CNN model for detecting lung cancer nodules	LIDC-IDRI	Accuracy: 92% Sensitivity: 93% Specificity: 92%	[81]
Tumor Staging			
evaluate the value of a nomogram integrating intratumoural and peritumoural features	199	AUC Training cohort: 0.81 Internal validation: 0.79 External validation: 0.71 Nomogram AUC: 0.74	[82]
Develop a model for quantitatively predicting N2 lymph node metastasis in presurgical clinical stage I-II NSCLC	140	Combined models (Rad + DL + Clinical) 0.88	[83]
Develop a deep learning (DL) signature for predicting lymph node metastasis	612	DL signature based on Swin Transformer 0.948–0.961	[84]
Predict the lymph node (N-) staging using traditional contrast-enhanced CT	100	AUC: 0.871	[85]

**Table 3:** The current status of multimodal research in radiogenomics.

Datasets	Genomicss Techniques	Result	References
75	EGFR	Recognize precisely epidermal growth factor receptor (EGFR) mutation in lung cancer patients AUC based on 18F-MPG PET: 0.94877 for primary cancers 0.91775 for metastasis cancers Based on 18F-FDG PET images: 0.87374 for primary lung cancers 0.82251 for metastasis	[111]
162	EGFR T790M resistance mutation	Predict the risk of developing EGFR T790M resistance mutation in metastatic NSCLC: AUC 0.853	[112]
204	STAS prediction	Establish a prediction model for spread through air spaces in early-stage NSCLC AUC based on mixed model: 0.85	[113]
57	Radiomic-based prediction of genetic alterations	Investigate the predictive capacity of radiomic for genetic mutations Associations identified between radiomic features and mutations	[114]
274	Glycolysis-related	Developed DeepGR, integrating CT radiomic and clinical features 0.8442 for glycolysis status classification	[115]
165	NGS, ctDNA analysis	Developed a radiomic model to predict progression-free survival C-index: 0.764	[116]
309	EGFR, KRAS, and ALK mutations	Developed a Radiomic Score to predict EGFR, KRAS, and ALK mutations and Overall Survival (OS) AUC: EGFR (0.86), KRAS/ALK (0.61–0.65) OS prediction models: C-index 0.63 (clinical), 0.79 (radiomic), 0.80 (clinical-radiomic)	[117]
247	NGS	Developed a multimodal biomarker for predicting immunotherapy response CT radiomic model: AUC = 0.65 PD-L1 IHC-texture model: AUC = 0.62 Pathologist-assessed: AUC = 0.73 Multimodal model: AUC = 0.80 Tumor mutational burden: AUC = 0.61	[12]
351	gene expression, gene mutation, or molecular subtypes	Gene masking was used to derive the learned associations Histology: AUC 0.86 Squamous cell: AUC 0.91	[118]
157	KRAS, TP53 and EGFR	M1 radiomic + clinical were 0.61 (TP53), 0.61 (KRAS) and 0.80 (EGFR) M2 radiomic + clinical were 0.64 (TP53), 0.62 (KRAS) and 0.81 (EGFR)	[119]

## Data Availability

All datasets utilize publicly available datasets based on image data from the TCIA database, including TCGA-LUAD, TCGA-LUSC, CPTAC-LUAD, and CPTAC-LSCC.
